# Diagnostic value of combination of biomarkers for malignant pleural mesothelioma: a systematic review and meta-analysis

**DOI:** 10.3389/fonc.2023.1136049

**Published:** 2023-04-11

**Authors:** Mucheng Zhu, Zhenhua Lu, Hao Guo, Xiaoting Gu, Defang Wei, Zhengyi Zhang

**Affiliations:** ^1^ Lanzhou University Second Hospital, Lanzhou University, Lanzhou, China; ^2^ School of Basic Medical Sciences, Lanzhou University, Lanzhou, China; ^3^ School of Medicine, Xiamen University, Xiamen, China

**Keywords:** MPM, diagnostic, combination of DNA and protein, meta-analysis, bioinformatics analysis, prognosis

## Abstract

**Introduction:**

Early-stage accurate diagnosis of malignant pleural mesothelioma (MPM) has always been a formidable challenge. DNA and protein as biomarkers for the diagnosis of MPM have received considerable attention, and yet the outcomes are inconsistent.

**Methods:**

In this study, a systematic search employing PubMed, EMBASE, and Cochrane Library to identify relevant studies from the first day of databases to October 2021. Moreover, we adopt the QUADAS-2 to evaluate the quality of eligible studies and Stata 15.0 and Review Manager 5.4 software programs to perform the meta-analysis. Additionally, bioinformatics analysis was performed at GEPIA for the purpose of exploring relationship between related genes and the survival time of MPM patients.

**Results:**

We included 15 studies at the DNA level and 31studies at the protein level in this meta-analysis. All results demonstrated that the diagnostic accuracy of the combination of MTAP + Fibulin-3 was the highest with the SEN 0.81 (95% CI: 0.67, 0.89) and the SPE 0.95 (95% CI: 0.90, 0.97). And the bioinformatics analysis indicated that the higher MTAP gene expression level was beneficial to enhance the survival time of MPM patients.

**Discussion:**

Nonetheless, as a result of the limitations of the included samples, it may be necessary to conduct additional research before drawing conclusions.

**Systematic review registration:**

https://inplasy.com/inplasy-2022-10-0043/, identifier INPLASY2022100043.

## 1 Introduction

Malignant pleural mesothelioma (MPM) is an aggressive malignancy that arises from the serosa of the body cavity. The majority of mesothelioma originates in the peritoneum, with 85-90 percent originating in the pleura (1). And the diagnostic standard methods of MPM include chest X-ray, computed tomography (CT) scan of chest and upper abdomen, examination of the pleural effusion using thoracentesis as well as histopathological examination with thoracoscopy. Furthermore, chest X-ray and chest CT lack sufficient sensitivity to diagnosis. And substantial volumes of pleural effusions can mask pleural/chest lesions as well as render undetectable a small amount of malignant pleural effusion. The gold standard for diagnosis of MPM is pathological examination. Nonetheless, as a result of inconspicuous clinical features and the long incubation period of MPM, patients are frequently diagnosed at an advanced stage, which is very unfavorable for treatment and prognosis. In this instance, additional techniques are required to demonstrate the lesions’ malignant biological potential (2).

Currently, due to their non-invasive nature and relatively low cost, tumor biomarkers for disease diagnosis have become increasingly desirable. In addition to many protein markers in MPM [mesothelin (MSLN), soluble mesothelin-related peptide **(**SMRP), osteopontin, fibrin, High Mobility Group Box 1 (HMGB1) protein, etc.], the DNA is released and expressed in cells (3–7). In this context, epigenetic markers such as DNA are emerging as promising biomarkers for multiple cancer types, including MPM (8, 9). Many scholars have published studies on DNA as biomarkers for early diagnosis of MPM. Moreover, it has been proved to be effective in recognizing the malignant transformation of tumors and in predicting prognosis in many cancers. It is worth mentioning that all DNA found in biological samples is stable and quantitative, which offers a significant benefit for laboratory detection.

All of the aforementioned factors support the need to identify appropriate biomarkers that can be easily measured in easily accessible tissues for early detection and improved prognosis. To date, no biomarker has been clinically available to diagnose MPM alone, and there are frequent cases of poor sensitivity (SEN) or specificity (SPE). Consequently, we constantly presume that seeking a combination of highly accurate diagnostics is a reasonable choice. In our previous study, diagnostic accuracy analysis was performed at the protein level. The results demonstrated that the Fibulin-3, pooled SEN 0.90 (95% CI: 0.74, 0.97) and SPE 0.91 (95% CI: 0.84, 0.95), might be a more appropriate indicator for early diagnosis of MPM comparing with the other two biomarkers (MSLN and SMRP) (10).

Encouragingly, genes such as BRCA1-associated protein 1 (BAP1), Methylthioadenosine (MTAP) and CDKN2A have been clinically applied as biomarkers for the early diagnosis of MPM (11). In this study, hence, we centered on BAP1, MTAP, and BAP1+MTAP, and in addition, combined them with MSLN, SMRP, and Fibulin-3 separately to analyze the diagnostic accuracy for MPM. Subsequently, we have obtained satisfactory results after the unified integration of protein markers and DNA for diagnostic accuracy evaluation, which implies that in the early diagnosis of MPM biomarkers, we can try to use more precise biomarker combinations. On top of this foundation, we conducted a bioinformatics study employing Gene Expression Profiling Interactive Analysis to assess the relationship between BAP1, MTAP and prognostic survival of MPM.

## 2 Materials and methods

This meta-analysis followed the Systematic Review and Meta-Analysis Guidelines (PRISMA) and was registered on the INPLASY (registration number: INPLASY2022100043). The registration information can be viewed in its entirety on inplasy.com (https://inplasy.com/inplasy-2022-10-0043/; accessed on 12 October 2022).

### 2.1 Search strategy and study selection

**Figure 1:**
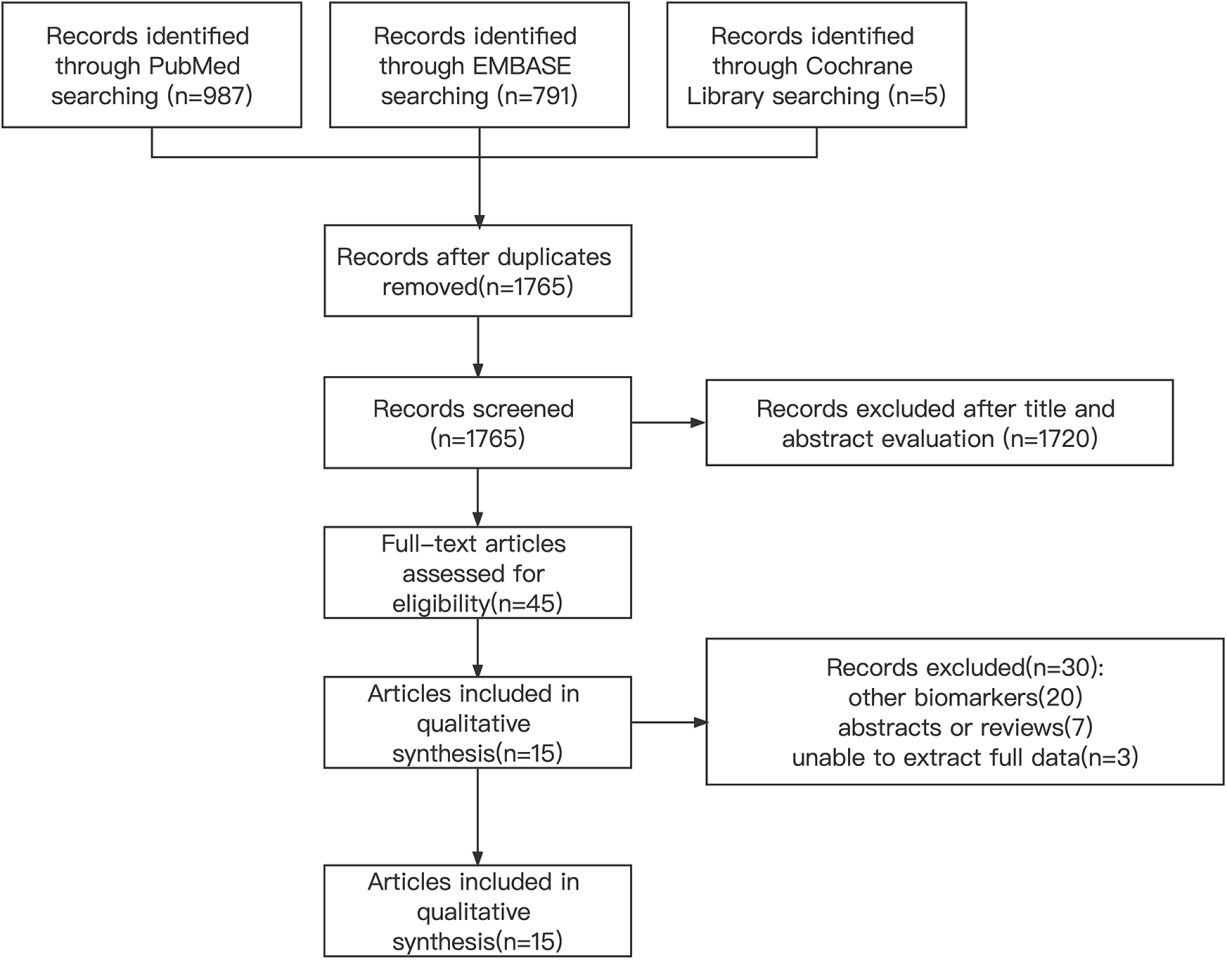
Flowchart of literature search.

We conducted systematic searches in PubMed, Embase and Cochrane libraries until October 2021. And the details of the literature search strategy are listed in **Figure 1** and **Table 1**. Furthermore, we sought references to relevant systematic reviews/meta-analyses to identify other potential studies. Protein biomarker information can be found in the corresponding citations (10).

**Table 1:** Summary of included studies.

First author	Year	MPM	non-MPM	Biomarker names	Reference test	characteristic	TP	FP	FN	TN
Zarah Glad Zimling (12)	2012	99	39	MTAP	Pathologic Diagnosis	NR	70	4	29	35
Sheffield BS (9)	2015	26	49	BAP1	Pathologic Diagnosis	Loss or deleted	7	0	19	49
Cigognetti M (13)	2015	212	65	BAP1	Pathologic Diagnosis	Loss or deleted	139	6	73	59
		319	65				137	6	182	59
		13	65				2	6	11	59
		45	42				29	6	16	36
Hida T (14)	2016	30	30	BAP1	Pathologic Diagnosis	Loss or deleted	27	0	3	30
Carbone M (15)	2016	26	43	BAP1	Pathologic Diagnosis	Loss or deleted	22	0	4	43
Hida T (8)	2017	51	25	BAP1	Pathologic Diagnosis	Loss or deleted	31	0	20	25
Pillappa R (16)	2017	10	10	BAP1	Histopathological examination	NR	3	0	7	10
Yoshimura M (17)	2017	41	258	BAP1	Histopathological examination	NR	23	5	18	253
Yoshiaki Kinoshita(18)	2018	12	17	MTAP	Pathologic Diagnosis	NR	10	0	2	17
				BAP1			4	0	8	17
				BAP1			27	0	18	21
Masayo Yoshimura (19)	2019	38	29	BAP1	Histopathological examination	Retained	20	0	18	29
				MTAP		Retained	18	0	20	29
David B. Chapel (20)	2020	99	20	MTAP	Histopathological examination	Loss or deleted	77	1	22	19
Yoshiaki Kinoshita (21)	2020	47	27	MTAP	Pathologic Diagnosis	Loss or deleted	33	0	14	27
				BAP1		Loss or deleted	27	0	20	27
				BAP1/MTAP		Loss or deleted	42	0	5	27
Kyra B. Berg (22)	2020	21	15	MTAP	fluorescence *in situ* hybridization	Loss/Retained	7	0	14	15
Hiroshima K (23)	2020	21	5	MTAP	immunohistochemistry+ fluorescence in situ hybridization effusion	Loss/Retained	16	0	5	5
Yoshimura M (24)	2020	40	20	MTAP	immunohistochemistry+ fluorescence in situ hybridization effusion	Loss/Retained	22	0	18	20

### 2.2 Inclusion and exclusion criteria

Studies meeting the following inclusion criteria were considered eligible for selection: (a) Study type: We evaluated the diagnostic precision of MPM antibody markers prospectively or retrospectively. (b) Participants: included patients diagnosed with MPM by histopathological examination, excluding patients with distant metastasis of MPM. (c) Reference standard: utilization of surgically obtained pleural biopsies in histopathological examination. (d) Results: area under the curve (AUC), SEN, SPE, diagnostic odds ratio (DOR).

Exclusion criteria: (a) papers in languages other than English and Chinese; (b) animal experiments; (c) papers about reviews, meta-analyses, conference summaries; case reports, letters, expert opinions; duplicates or multiple publications. (d) insufficient data to calculate SEN and SPE.

### 2.3 Selection of studies

Two authors independently screened the titles and abstracts of each study following the completion of the search. And we obtained all articles deemed appropriate on either side of the full text for further evaluation. The same two authors would evaluate potential full-text and select studies for a discussion of inclusion on the basis of inclusion/exclusion criteria and reach an agreement through discussion and consensus to resolve distinctions. And a third reviewer will be sought if an agreement cannot be reached.

### 2.4 Data characteristics

Two independent reviewers were tasked with conducting a literature search and assessing the applicability of each study based on the inclusion criteria. During the same time, a third researcher resolved the conflict problems if they existed.

Following a review of the full texts of the included articles, we compiled the following information: (a) Study information: authors, year of publication, the language of publication, information of journal and type of study; (b) Sample size: number of MPM patients and non-MPM patients; (c) Index test: detection methods and types of biomarkers; (d) Baseline data: age, gender and diagnosis; (e) Number of outcomes: true positive(TP), false positive(FP), true negative(TN) and false negative(FN) for each study.

### 2.5 Risk of bias and quality assessment of evidence

We adopted the revised The Quality Assessment of Diagnostic Accuracy Studies 2 quality assessment tool (QUADAS-2) (HTA Program 2011 (www.hta.ac.uk)) with the aim of assessing the quality of each study. This tool includes 4 predominant areas that discuss patient selection, index test, reference standards, flow and timing. And the risk of bias was assessed by the results of 4 domains and each question was answered as “yes”, “no” or “unclear”. The applicability concerns were assessed by the results of the first 3 domains and each question was rated as “low,” “high,” or “unclear” (25). This evaluation was done independently by two reviewers.

### 2.6 Assessment of publication bias

We utilized Deek’s funnel plot to detect publication bias. And data with severe publication bias (P<0.05) were excluded.

### 2.7 Survival analyses and RNA-seq data acquisition

We performed an overall prognostic analysis of the BAP1 and MTAP gene in mesothelioma on GEPIA (GEPIA: a web server for cancer and normal gene expression profiling and interactive analyses. Nucleic Acids Res, 10.1093/nar/gkx247). In this database, the RNA-seq data of 86 tumor tissues and clinical information of patients including age, gender, tumor stage and histologic subtype (epithelioid, sarcomatous, biphasic) were collected. We divided the patients into a high-expression group and a low-expression group according to the median expression level of the MTAP gene to evaluate the prognosis of the MPM patients. Kaplan–Meier (KM) survival analyses were conducted using the R package (survminer, v.0.4.9 and survival, v.3.2.10) (https://CRAN.R-project.org/package=survminer) (http://cran.r-project.org/package=survival).

### 2.8 Statistical analysis

We adopted Stata 15.0 (Stata Corporation, College Station, TX, USA) and Review Manager 5.4 statistical software programs for the purpose of processing the data and detecting the heterogeneity of the studies in this meta-analysis.

Subsequently, we extracted true TP, FP, TN and FN data from each study and obtained a 2x2 contingency table. Besides, the SEN, SPE, positive likelihood ratio (PLR), negative likelihood ratio (NLR) and DOR for each study were calculated to generate a ROC curve with STATA software. The resulting regression coefficients were used to fit the ROC curves, yielding the AUC, SEN, SPE, and likelihood ratios (LRs). The RNA-Seq datasets GEPIA used are based on the UCSC Xena project (http://xena.ucsc.edu), which are computed by a standard pipeline. The statistical calculations of data from TCGA were processed through R software (v.3.6.3).

## 3 Results

### 3.1 The characteristic of included studies

The included studies were published between 2008 and 2020. We collected data on 1054 MPM patients and 810 non-MPM patients. After eliminating duplicate articles, reviewing titles and abstracts, we conducted a full-text screening of the remaining 45 studies, and eventually determined to include 15 studies. The details of the literature search flowchart are illustrated in **Figure 1**. Moreover, the characteristics of the included studies are summarized in **Table 1**. All MPM patients are diagnosed by pathological examination and the results contain biomarkers MTAP or BAP-1.

### 3.2 Quality assessment

QUADAS-2 has been used to evaluate the methodological quality of studies. **Supplementary Figure 1** in the Appendix summarizes the quality of included studies. Whereas **Supplementary Figure 2** provides details on the risk of bias and applicability concerns for each included study.

### 3.3 Diagnostic accuracy

15 studies (9, 12–24) assessed the diagnostic value of the combination of biomarkers for the diagnosis of MPM. There were a total of 1864 patients included.

**Figure 2:**
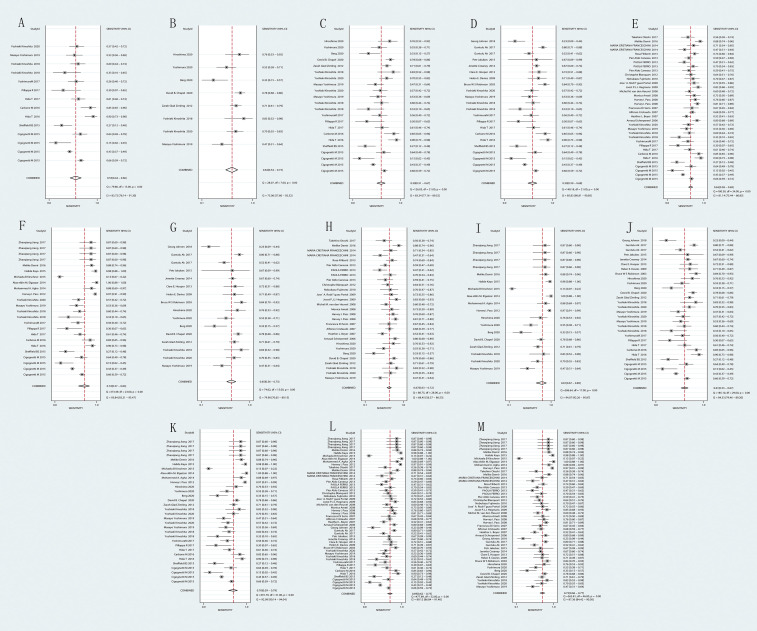
Forest plot for the pooled sensitivity of biomarkers. **(A)** BAP1; **(B)** MTAP; **(C)** BAP1+MTAP. **(D)** BAP1+MSLN; **(E)** BAP1+SMRP; **(F)** BAP1+ Fibulin-3; **(G)** MTAP+ MSLN; **(H)** MTAP+ SMRP; **(I)** MTAP+ Fibulin-3; **(J)** BAP1+MTAP+MSLN; **(K)** BAP1+MTAP+ Fibulin-3; **(L)** BAP1+MSLN+SMRP+Fibulin-3; **(M)** MTAP+MSLN+SMRP+Fibulin-3.

**Figure 3:**
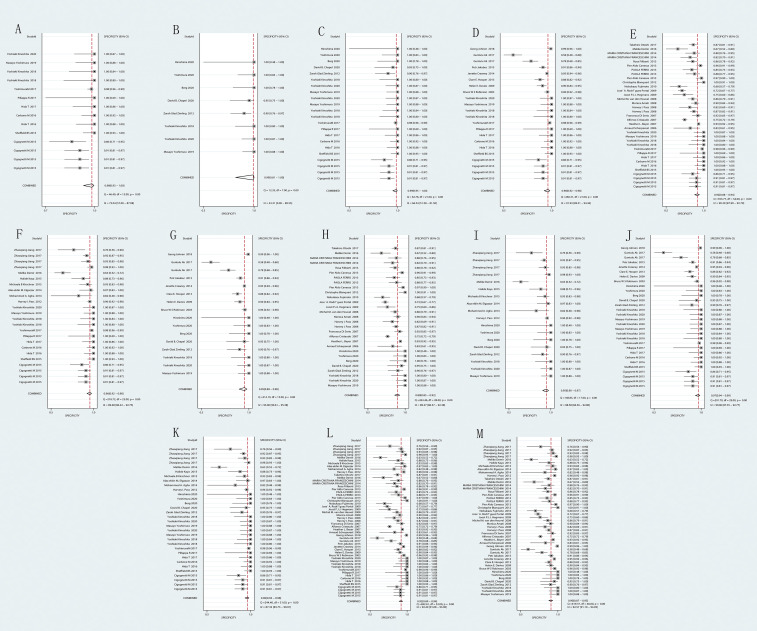
Forest plot for the pooled specificity (SPE) of biomarkers. **(A)** BAP1; **(B)** MTAP; **(C)** BAP1+MTAP. **(D)** BAP1+MSLN; **(E)** BAP1+SMRP; **(F)** BAP1+ Fibulin-3; **(G)** MTAP+ MSLN; **(H)** MTAP+ SMRP; **(I)** MTAP+ Fibulin-3; **(J)** BAP1+MTAP+MSLN; **(K)** BAP1+MTAP+ Fibulin-3. **(L)** BAP1+MSLN+SMRP+Fibulin-3; **(M)** MTAP+MSLN+SMRP+Fibulin-3.

**Figure 4:**
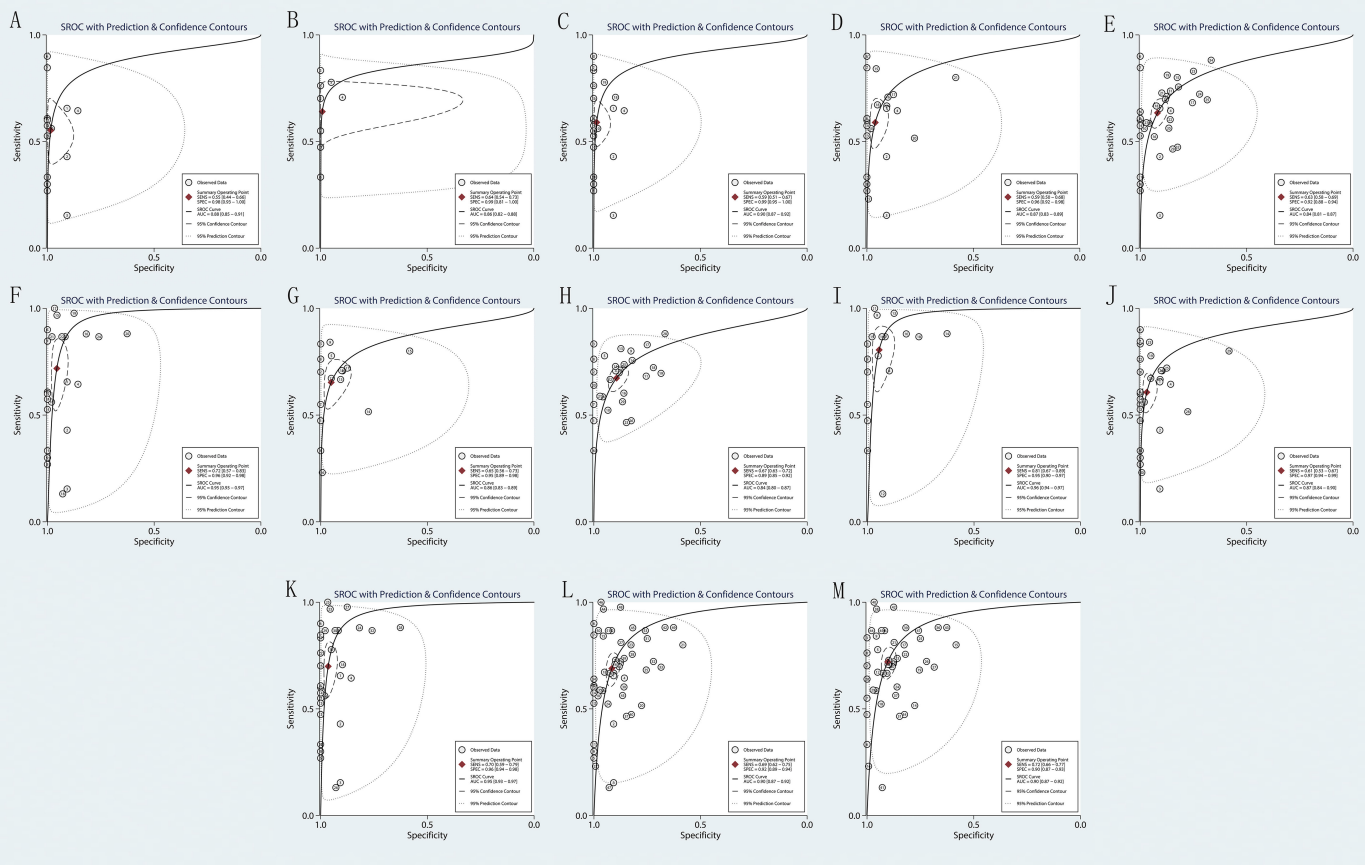
Forest plot for the area under the curve (AUC) of biomarkers. **(A)** BAP1; **(B)** MTAP; **(C)** BAP1+MTAP. **(D)** BAP1+MSLN; **(E)** BAP1+SMRP; **(F)** BAP1+ Fibulin-3; **(G)** MTAP+ MSLN; **(H)** MTAP+ SMRP; **(I)** MTAP+ Fibulin-3; **(J)** BAP1+MTAP+MSLN; **(K)** BAP1+MTAP+ Fibulin-3. **(L)** BAP1+MSLN+SMRP+Fibulin-3; **(M)** MTAP+MSLN+SMRP+Fibulin-3.

Forest plots from the meta-analysis illustrated that the pooled sensitivity of the combination of MTAP + Fiblin-3 for MPM diagnosis was 0.81 (95% CI, 0.67–0.89) and the pooled specificity was 0.95 (95% CI, 0.90–0.97). The AUC was 0.96 (95% CI: 0.94-0.97). The data for the diagnosis of MPM by the DNA and combinations of DNA and protein are detailed in **Figures 2**, **3**. The SROC curves are given in **Figure 4**. And the full data results of our analysis are indicated in **Table 2**.

**Table 2:** Summary of results of meta-analysis.

Biomarker	Sensitivity(95% CI)	Specificity(95% CI)	AUC(95% CI)
BAP1	0.55[0.44-0.66]	0.98[0.93-1.00]	0.88[0.85-0.91]
MTAP	0.64[0.54-0.73]	0.99[0.81-1.00]	0.86[0.82-0.88]
BAP1+MTAP	0.59[0.51-0.67]	0.99[0.95-1.00]	0.90[0.87-0.92]
BAP1+MSLN	0.59[0.59-0.68]	0.96[0.92-0.98]	0.87[0.83-0.89]
BAP1+SMRP	0.63[0.58-0.69]	0.92[0.88-0.94]	0.84[0.81-0.87]
BAP1+Fibulin-3	0.72[0.57-0.83]	0.96[0.92-0.98]	0.95[0.93-0.97]
MTAP+MSLN	0.65[0.56-0.73]	0.95[0.89-0.98]	0.86[0.83-0.89]
MTAP+SMRP	0.67[0.63-0.72]	0.89[0.85-0.92]	0.84[0.80-0.87]
MTAP+Fibulin-3	0.81[0.67-0.89]	0.95[0.90-0.97]	0.96[0.94-0.97]
BAP1+MTAP+MSLN	0.61[0.53-0.67]	0.97[0.94-0.99]	0.87[0.84-0.90]
BAP1+MTAP+Fibulin-3	0.70[0.59-0.79]	0.96[0.94-0.98]	0.95[0.93-0.97]
BAP1+MSLN+SMRP+Fibulin-3	0.69[0.62-0.75]	0.92[0.89-0.94]	0.90[0.87-0.92]
MTAP+MSLN+SMRP+Fibulin-3	0.72[0.66-0.77]	0.90[0.87-0.93]	0.90[0.87-0.92]

BAP1: BRCA1-associated protein 1; MTAP: methylthioadenosine; MSLN:mesothelin; SMRP: soluble mesothelin-related peptide.

### 3.4 Publication bias

The Deek’s funnel plot asymmetry test was applied to evaluate studies for potential publication bias (**Supplementary Figure 3**). These results indicate that the research articles included in this meta-analysis have no publication bias.

### 3.5 Prognostic analysis of BAP1 and MTAP gene in mesothelioma

**Figure 5:**
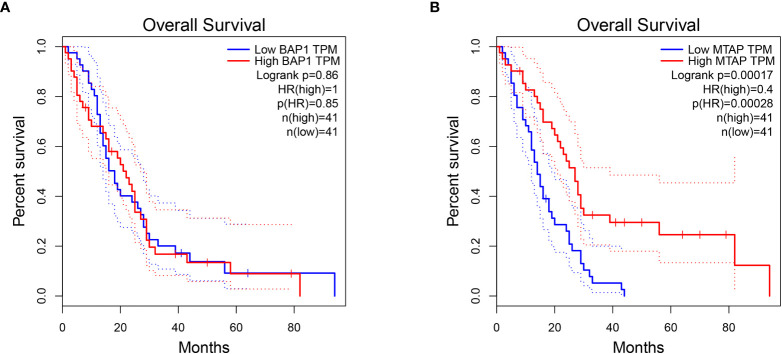
Overall prognostic analysis of BAP1 **(A)** gene and MTAP **(B)** gene in mesothelioma.

The overall prognosis of BAP1 and MTAP genes in mesothelioma was analyzed on GEPIA. And the Log-rank test demonstrated that the survival time of the MTAP high-expression group and low-expression group was statistically different (p = 0.00017). And the results suggested that higher expression of the MTAP gene was in association with longer survival time in MPM patients. The correlation between BAP1 gene expression and MPM prognosis was not significant. The results are displayed in **Figure 5**.

### 3.6 Subgroup analysis

**Figure 6:**
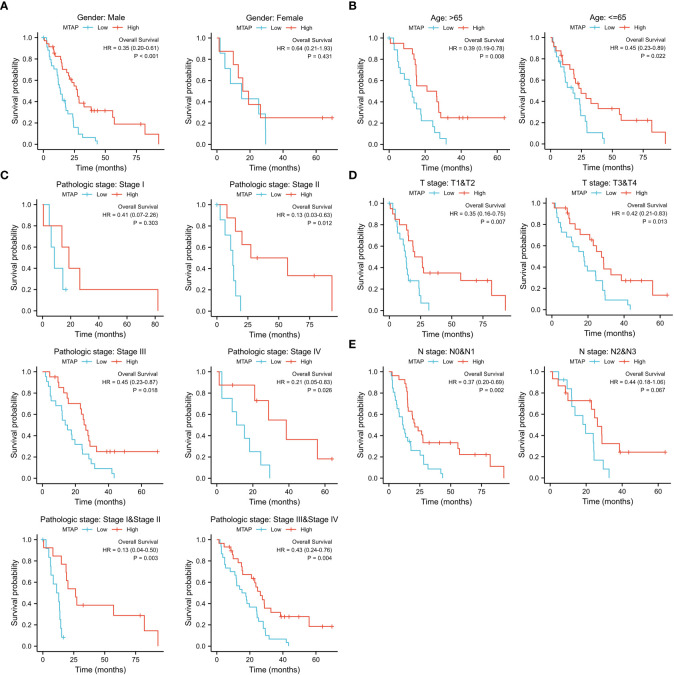
Subgroup prognostic analysis of MTAP gene in mesothelioma. **(A)**: Gender group; **(B)**: Age group; **(C)**: Pathological stage; **(D)**: Primary tumor stage (T stage); **(E)**: MPM regional lymph node metastasis stage (N stage).

**Figure 7:**
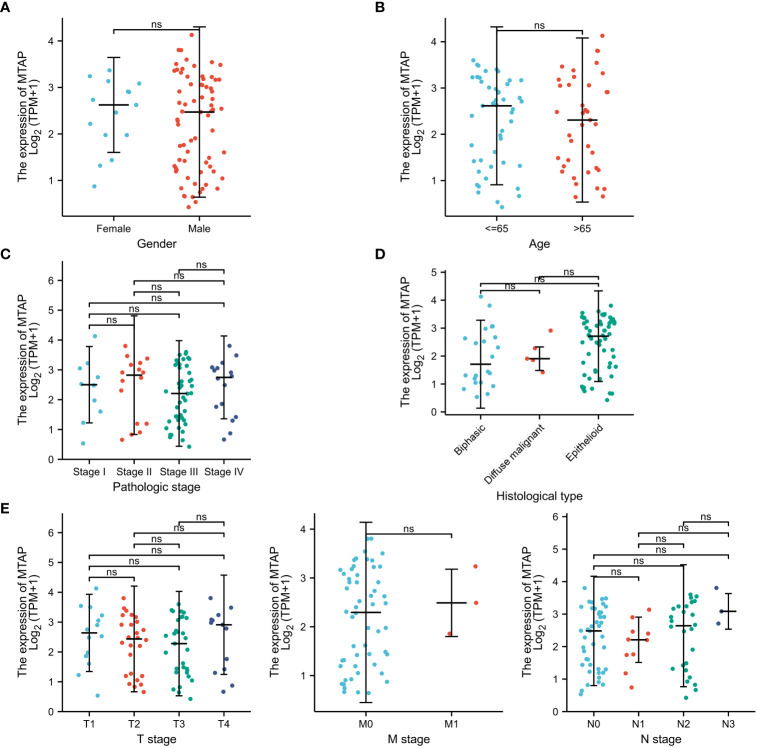
Subgroup differences of MTAP gene expression in mesothelioma. ns, not significant.

The results of the subgroup analysis demonstrated that in male MPM patients, higher MTAP gene expression was associated with longer survival time (P < 0.001) (**Figure 6A**). MTAP gene indicated that higher expression corresponded to longer survival time at all ages (**Figure 6B**). Stage I, Stage II, Stage III, Stage I & Stage II, and Stage II & Stage IV all revealed the same trend, higher MTAP expression is associated with longer survival time in MPM patients (P<0.05) (**Figure 6C**). In T1& T2, T3&T4 and N0&N1 subgroups, the higher the MTAP expression, the longer the survival time (P<0.05) (**Figure 6D**). In the Pathologic stage (Stage I) and N stage (N2&N3), MTAP expression and patient survival time did not show a correlation (**Figure 6E**). As shown in **Figure 7**, there was no significant difference in MTAP expression among all subgroups of MPM patients.

## 4 Discussion

Malignant pleural mesothelioma is the most common type of primary pleural tumor. Despite the fact that the histologic diagnosis of MPM is currently the most widely used in clinical practice, patients will have a better prognosis with an early diagnosis. Currently, there are no reliable indicators for longitudinal surveillance and associated risk assessment of asbestos-exposed populations (26). It is widely known that the development of non-invasive diagnostic methods for oncology is a major challenge in modern oncology, and the analysis of samples of plasma, serum, urine, cerebrospinal fluid and pleural fluid is a suitable method to identify markers in association with cancer progression, as these samples are easier to collect and less invasive to the patient. Ideally, Biomarkers for cancer detection should ideally be readily available and inexpensive to measure, allowing for early disease detection and an improved prognosis (27). Meanwhile, many studies have identified changes in DNA expression in tumor tissues and body fluids from various tumor pathological processes, suggesting DNA as a potential diagnostic marker, Accordingly, our research focuses on whether DNA or combinations of DNA and other biomarkers can be the most appropriate solution for early diagnosis of MPM. Moreover, the gold standard for clinical diagnosis of MPM is essential in the final confirmation of diagnosis (28–30).

The purpose of this study is to evaluate the diagnostic value of DNA as well as multiple marker combinations for MPM using a meta-analysis approach. And the studies we included were free of publication bias, indicating that the results of this study are reliable. Comparing the results between the 13 groups revealed that MTAP+Fibulin-3 had a better specificity as well as AUC, but the sensitivity was not so outstanding, with a sensitivity and specificity of 0.81 (95% CI: 0.67, 0.89) and 0.95 (95% CI: 0.90,0.97), respectively. The bioinformatic analysis demonstrated that MPM patients with higher MTAP gene expression had had a longer period of survival. All of the aforementioned findings indicate that we can attempt to increase the survival time of MPM patients by regulating the expression of MTAP. Research from both early diagnosis and improved prognosis will be more beneficial to the whole process of MPM treatment. Except for that, our findings make some efforts in different levels of binding (DNA and protein combination) for diagnosis, and yet due to the low sensitivity of MTAP+Fibulin-3, which may be caused by variables including the amount of data included in the study, the diagnostic effect of BAP1, MTAP, as well as other combinations are not outstanding, so we believe that in practical clinical applications we can preferentially recommend Fibulin-3 as a biomarker for early diagnosis of MPM since it is more readily available as a protein and can be preserved until the assay is completed, and our team has published the diagnostic accuracy of Fibulin-3 in previous studies (10).

In our previous study, Fublin-3 can be detected in plasma and pleural effusion, which can be used as a biomarker for early diagnosis. However, due to the lack of specificity, for MPM with unclear diagnosis, MTAP can be further detected by immunohistochemistry to improve the specificity of diagnosis. At the same time, MTAP can affect the prognosis of patients with MPM according to the results of bioinformatics analysis, which can help to guide the prognosis and treatment of MPM patients. What’s more, we also determined that it was not easy to obtain pleural effusion and tissue specimens of suspected MPM patients in the early stage, which may increase the difficulty of our early diagnosis. More research is required to determine whether MTAP can be extracted from plasma.

The results of this study may aid in the early clinical diagnosis of MPM: if Fibulin-3 can be detected preferentially, it will be the most recommended biomarker, and other markers at the DNA level are not recommended preferentially. Nonetheless, considering the excellent specificity of MTAP+Fibulin-3, we suggest that future clinical studies on MTAP could be more in-depth, to compensate for the low sensitivity of MTAP+Fibulin-3 in the diagnosis of MPM due to factors including the amount of data or distinctions in laboratory techniques, which may be a new breakthrough point for the early diagnosis of MPM in the future. Meanwhile, there are some limitations of this study. Although 15 studies were included, the small number of sample studies might compromise the reliability of the pooled estimates of the meta-analysis. In addition, the majority of studies did not report the time interval between diagnostic testing and reference standard testing. Most of the included studies were cross-sectional studies involving patients with advanced disease, and there may be inconsistent laboratory methods and technical irregularities, which largely limit the diagnostic accuracy of MPM biomarkers.

## 5 Conclusions

In conclusion, multiple normalizers may be more appropriate than a single reference DNA for obtaining reliable data. Increasing the expression of the MTAP gene can well enhance the prognosis and prolong the survival time of MPM patients. Such exploration can help MPM early diagnosis and improve prognosis to move faster towards precision medicine.

## Data availability statement

The datasets presented in this study can be found in online repositories. The names of the repository/repositories and accession number(s) can be found in the article/**Supplementary Material**.

## Author contributions

MZ and ZZ provided concept, design, and manuscript preparation. ZL developed the search strategies, conducted literature and study selection. HG and MZ contributed to the development of selection criteria and the risk of bias assessment strategy. XG and DW extracted data from the included studies and assessed the risk of bias and summarize the evidence. MZ and ZL read, provided feedback, and approved the final manuscript. ZZ contributed to review and funding acquisition. All authors contributed to the article and approved the submitted version.
