# Supplementary material for: Diagnostic value of combination of biomarkers for malignant pleural mesothelioma: a systematic review and meta-analysis
Source: Front Oncol. 2023 Apr 11;13:1136049. doi: 10.3389/fonc.2023.1136049 (PMC10126368; doi:10.3389/fonc.2023.1136049)
Supplement: Supplementary file 1 [file DataSheet_1.pdf]

Supplementary Material

Diagnostic Value of Combination of Biomarkers for Malignant Pleural Mesothelioma: A Systematic Review and Me-ta-Analysis

Mucheng Zhu, Zhenhua Lu, Hao Guo, Xiaoting Gu, Defang Wei, Zhengyi Zhang*

*** Correspondence:** zhangzhengyi11@lzu.edu.cn

1 Searching strategy

PubMed

#1 "Sensitivity AND Specificity"[Mesh] OR "False Positive Reactions"[Mesh] OR "False Negative Reactions"[Mesh] OR "ROC Curve"[Mesh] OR "Predictive Value of Tests"[Mesh] OR sensitivity[Title/Abstract] OR specificity[Title/Abstract] OR receiver operating characteristic[Title/Abstract] OR receiver operator characteristic[Title/Abstract] OR predictive value*[Title/Abstract] OR roc[Title/Abstract] OR pre-test odds[Title/Abstract] OR pretest odds[Title/Abstract] OR pre-test probability*[Title/Abstract] OR pretest probability*[Title/Abstract] OR post-test odds[Title/Abstract] OR posttest odds[Title/Abstract] OR post-test probabilit*[Title/Abstract] OR posttest probabilit*[Title/Abstract] OR likelihood ratio*[Title/Abstract] OR positive predictive value*[Title/Abstract] OR negative predictive value*[Title/Abstract] OR false negative*[Title/Abstract] OR false positive*[Title/Abstract] OR true negative*[Title/Abstract] OR true positive*[Title/Abstract] OR fn[Title/Abstract] OR fp[Title/Abstract] OR tn[Title/Abstract] OR tp[Title/Abstract]

#2 "Pleural Neoplasms"[MeSh] OR "mesothelioma, malignant"[MeSh]

#3 "malignant mesotheliomas"[Title/Abstract] OR "mpm"[Title/Abstract] OR "pleural mesothelioma"[Title/Abstract] OR "pleural cancer"[Title/Abstract] OR "pleural tumor"[Title/Abstract] OR "pleural neoplasms"[Title/Abstract]

#4 #2 OR #3

#5 "Biomarkers"[Mesh] OR "Biomarkers, Tumor"[Mesh] OR "biological marker"[Title/Abstract] OR "biological markers"[Title/Abstract] OR biomarker*[Title/Abstract]

#6 "Galectin-1"[Title/Abstract] OR "Mesothelin"[Title/Abstract] OR "Osteopontin"[Title/Abstract] OR "shed SDC-1"[Title/Abstract] OR "VEGF"[Title/Abstract] OR "MMP-7"[Title/Abstract] OR "HGF"[Title/Abstract] OR "TIMP-1"[Title/Abstract] OR "NRG1-Î²1"[Title/Abstract] OR "intelectin-1"[Title/Abstract] OR "CTC"[Title/Abstract] OR "miR-103"[Title/Abstract] OR "miR-126"[Title/Abstract] OR "miR132-3p"[Title/Abstract] OR "miR548a-3p"[Title/Abstract] OR "miR625-3p"[Title/Abstract] OR "lncRNARP1â€“86D1.3"[Title/Abstract] OR "DRAM1"[Title/Abstract] OR "ARSA"[Title/Abstract] OR "miR2053"[Title/Abstract] OR

"MPF"[Title/Abstract] OR "Circulating tumor cell"[Title/Abstract] OR "Megakaryocyte Potentiating Factor"[Title/Abstract] OR "EZH2"[Title/Abstract] OR "BAP1"[Title/Abstract] OR "MTAP"[Title/Abstract] OR "9p21"[Title/Abstract] OR "SMRPs"[Title/Abstract] OR "MicroRNAs"[Title/Abstract] OR "HMGB1"[Title/Abstract] OR "High Mobility Group Box 1"[Title/Abstract] OR "Aquaporin-1"[Title/Abstract] OR "Fibulin-3"[Title/Abstract] OR "EFEMP1"[Title/Abstract] OR "thrombomodulin"[Title/Abstract] OR "CD157"[Title/Abstract] OR "Secreted miRNAs"[Title/Abstract] OR "Tissue miRNAs"[Title/Abstract] OR "CYFRA 21-1"[Title/Abstract] OR "CEA"[Title/Abstract] OR "Midkine"[Title/Abstract] OR "TGF-Î²"[Title/Abstract] OR "MUC4"[Title/Abstract] OR "miRNA103"[Title/Abstract] OR "miR98"[Title/Abstract] OR "TRX"[Title/Abstract] OR "BAP1"[Title/Abstract] OR "SM"[Title/Abstract] OR "pOPN"[Title/Abstract] OR "MSLN"[Title/Abstract] OR "Ki-67"[Title/Abstract] OR "repp86"[Title/Abstract] OR "D2-40"[Title/Abstract] OR "HBME-1"[Title/Abstract] OR "MOC-31"[Title/Abstract] OR "TM"[Title/Abstract] OR "h-caldesmon"[Title/Abstract] OR "80HdG"[Title/Abstract] OR "TTF-1"[Title/Abstract] OR "BerEp4"[Title/Abstract] OR "N-ERC"[Title/Abstract] OR "CECs"[Title/Abstract] OR "Integrin-linked kinase"[Title/Abstract] OR "CCL2"[Title/Abstract] OR "galectin-3"[Title/Abstract] OR "GLUT-1"[Title/Abstract] OR "inflam-matory proteins"[Title/Abstract] OR "proliferative proteins"[Title/Abstract] OR "N-glycoproteins"[Title/Abstract] OR "THY1"[Title/Abstract] OR "CD90"[Title/Abstract] OR "te-neurin-2"[Title/Abstract] OR "ODZ2"[Title/Abstract] OR "survivin"[Title/Abstract] OR "Numb"[Title/Abstract] OR "C-ERC"[Title/Abstract] OR "miR-34b"[Title/Abstract] OR "methyla-tion"[Title/Abstract] OR "CD157"[Title/Abstract] OR "SPP1"[Title/Abstract] OR "Cldn3"[Title/Abstract] OR "cldn4"[Title/Abstract] OR "miR-197-3p"[Title/Abstract] OR "miR-1281"[Title/Abstract] OR "miR 32-3p"[Title/Abstract] OR "Macrophage migration inhibitory factor"[Title/Abstract] OR "MIF"[Title/Abstract] OR "CD74"[Title/Abstract] OR "syndecan-1"[Title/Abstract] OR "sgc-1"[Title/Abstract] OR "p16"[Title/Abstract] OR "CDKN2A"[Title/Abstract] OR "SP1"[Title/Abstract] OR "glycodelin"[Title/Abstract] OR "miR-31"[Title/Abstract] OR "miR-29c*"[Title/Abstract] OR "SOMAmer proteomic classifi-er"[Title/Abstract] OR "SOMAscan"[Title/Abstract] OR "CD44"[Title/Abstract] OR "HA"[Title/Abstract] OR "Hyaluronate"[Title/Abstract] OR "MOK gene"[Title/Abstract] OR "miR-548a-3p"[Title/Abstract] OR "miR-20a"[Title/Abstract] OR "BDNF"[Title/Abstract] OR "miR-143"[Title/Abstract] OR "miR-210"[Title/Abstract] OR "miR-200c"[Title/Abstract] OR "hsa-miR-2053"[Title/Abstract] OR "desmin"[Title/Abstract] OR "miR 34b methyla-tion"[Title/Abstract] OR "miR 34c methylation"[Title/Abstract] OR "circulating MPC"[Title/Abstract] OR "prosaposin"[Title/Abstract] OR "quiescin Q6 sulfhydryl oxidase 1"[Title/Abstract] OR "activin A"[Title/Abstract] OR "EMA"[Title/Abstract] OR "lncRNA"[Title/Abstract] OR "Wilms tumor pro-tein-1"[Title/Abstract] OR "WT1"[Title/Abstract] OR "B72.3"[Title/Abstract] OR "Leu-M1"[Title/Abstract] OR "Ber-EP4"[Title/Abstract] OR "HEA-125"[Title/Abstract] OR "anti-BGR A, B, H"[Title/Abstract] OR "ca19-9"[Title/Abstract] OR "CD30"[Title/Abstract] OR "Ber-H2"[Title/Abstract] OR "HMFG-2"[Title/Abstract] OR "SP-A"[Title/Abstract] OR "SP-B"[Title/Abstract] OR "CD15"[Title/Abstract] OR "CK20"[Title/Abstract] OR "CK7"[Title/Abstract] OR "BG8"[Title/Abstract] OR "CK5/6"[Title/Abstract] OR "p63"[Title/Abstract] OR "Membranous podoplanin"[Title/Abstract] OR "Cyfra 21.1"[Title/Abstract] OR "CD146"[Title/Abstract] OR "E-cadherin"[Title/Abstract] OR "CD90"[Title/Abstract] OR "PAX2"[Title/Abstract] OR "napsin A"[Title/Abstract] OR "claudin-4"[Title/Abstract] OR "miR-130a"[Title/Abstract] OR "miR-193a"[Title/Abstract] OR "miR-675"[Title/Abstract] OR "miR-141"[Title/Abstract] OR "miR-205"[Title/Abstract] OR "miR-375"[Title/Abstract] OR "miR-103a-3p"[Title/Abstract] OR "miR-30e-3p"[Title/Abstract] OR "nuclear 5-hmC"[Title/Abstract] OR

"MMP9"[Title/Abstract] OR "cathepsin B"[Title/Abstract] OR "C reactive protein"[Title/Abstract] OR "kallikrein 12"[Title/Abstract] OR "CA153"[Title/Abstract] OR "Pseudouridine"[Title/Abstract] OR "HEG1"[Title/Abstract] OR "SKM9-2"[Title/Abstract] OR "glypican-1"[Title/Abstract] OR "tenascin XB"[Title/Abstract] OR "TNXB"[Title/Abstract] OR "PD L1"[Title/Abstract] OR "NF2"[Title/Abstract] OR "CA125"[Title/Abstract] OR "miR-132-3p"[Title/Abstract] OR "miR-126-3p"[Title/Abstract] OR "disabled homolog 2"[Title/Abstract] OR "DAB2"[Title/Abstract] OR "mucin 21"[Title/Abstract] OR "MUC21"[Title/Abstract] OR "SOX6"[Title/Abstract] OR "PDIA6"[Title/Abstract] OR "MEG3"[Title/Abstract] OR "SDCCAG3"[Title/Abstract] OR "IGHG3"[Title/Abstract] OR "IGHG1"[Title/Abstract] OR "RASSF1"[Title/Abstract] OR "PGR1"[Title/Abstract] OR "ESR1"[Title/Abstract] OR "CDH1"[Title/Abstract] OR "APC"[Title/Abstract] OR "chitinase-3-like-1"[Title/Abstract] OR "YKL-40"[Title/Abstract] OR "VEGFbeta"[Title/Abstract] OR "miR-145"[Title/Abstract] OR "miR-652"[Title/Abstract] OR "gam-ma-catenins"[Title/Abstract] OR "BG-8"[Title/Abstract] OR "AST"[Title/Abstract] OR "mcm7"[Title/Abstract] OR "geminin"[Title/Abstract] OR "topo IIÎ±"[Title/Abstract] OR "Noxa"[Title/Abstract] OR "CL-4"[Title/Abstract] OR "ca549"[Title/Abstract] OR "IMP3"[Title/Abstract] OR "KOC"[Title/Abstract] OR "Met-TM"[Title/Abstract] OR "SSEA-1"[Title/Abstract] OR "ACE"[Title/Abstract] OR "CA19.9"[Title/Abstract] OR "vi-mentin"[Title/Abstract] OR "LDH"[Title/Abstract] OR "caveolin-1"[Title/Abstract] OR "Cav-1"[Title/Abstract] OR "CD138"[Title/Abstract] OR "rrr-r2"[Title/Abstract] OR "Calretinin"[Title/Abstract] OR "CR"[Title/Abstract] OR "CD24"[Title/Abstract] OR "maspin"[Title/Abstract] OR "p40"[Title/Abstract] OR "AE1"[Title/Abstract] OR "AE3"[Title/Abstract] OR "TPM"[Title/Abstract] OR "CLDN-15"[Title/Abstract] OR "miR-200a"[Title/Abstract] OR "miR-34a"[Title/Abstract] OR "PDL1"[Title/Abstract] OR "antigen 15-3"[Title/Abstract] OR "folic acid"[Title/Abstract] OR "vitamin B12"[Title/Abstract] OR "ferritin"[Title/Abstract] OR "TOP2A"[Title/Abstract] OR "miR-193a-3p"[Title/Abstract] OR "TLR3"[Title/Abstract] OR "galec-tin-1"[Title/Abstract] OR "EGFRAS1"[Title/Abstract] OR "FGF-9"[Title/Abstract] OR "Clau-din-5"[Title/Abstract] OR "cax"[Title/Abstract] OR "AMAD-2"[Title/Abstract] OR "WT49"[Title/Abstract] OR "ILK"[Title/Abstract] OR "miR-21"[Title/Abstract] OR "hsa-miR-29c*"[Title/Abstract] OR "miR-17*"[Title/Abstract] OR "miR-30"[Title/Abstract] OR "VOCs"[Title/Abstract] OR "P5"[Title/Abstract] OR "P25"[Title/Abstract] OR "P1"[Title/Abstract] OR "P3"[Title/Abstract] OR "P7"[Title/Abstract] OR "GAS5"[Title/Abstract] OR "DAKO"[Title/Abstract]

#7 #5 OR #6

#8 #1 AND #4 AND #7

2 Supplementary Figures

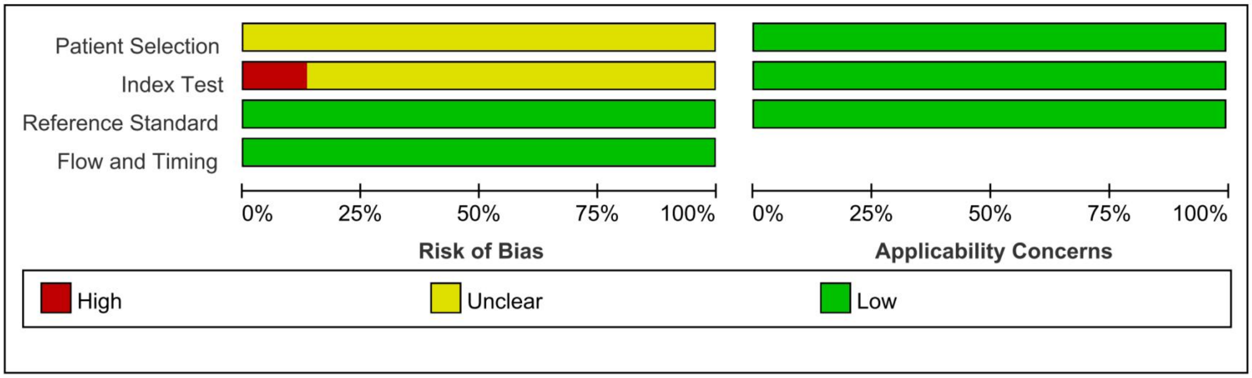

Supplementary Figure 1. The quality of included studies.

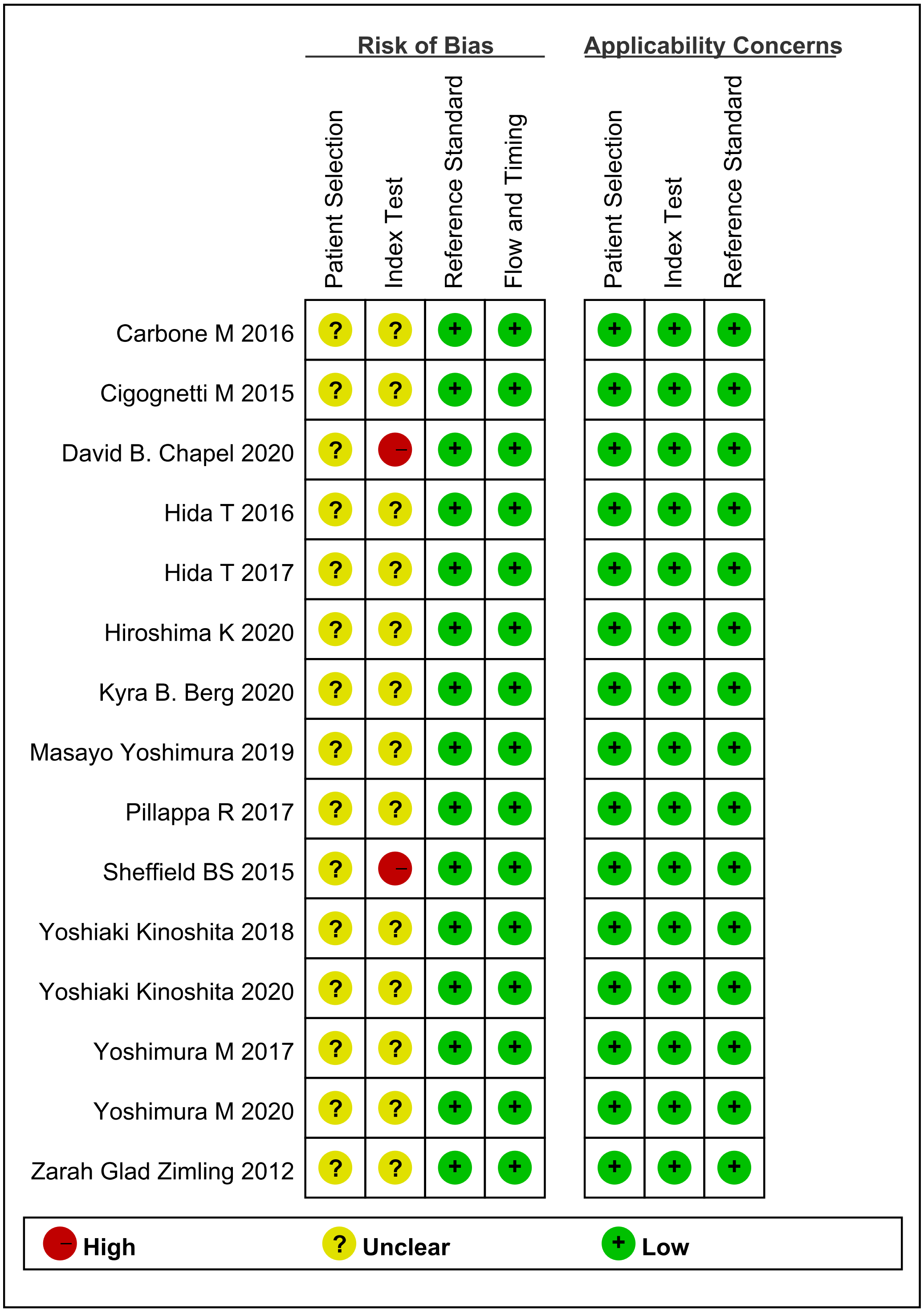

Supplementary Figure 2. The detailed information about the risk of bias and applicability concerns for each included study Quality plot graphically representing the risk of bias analysis.

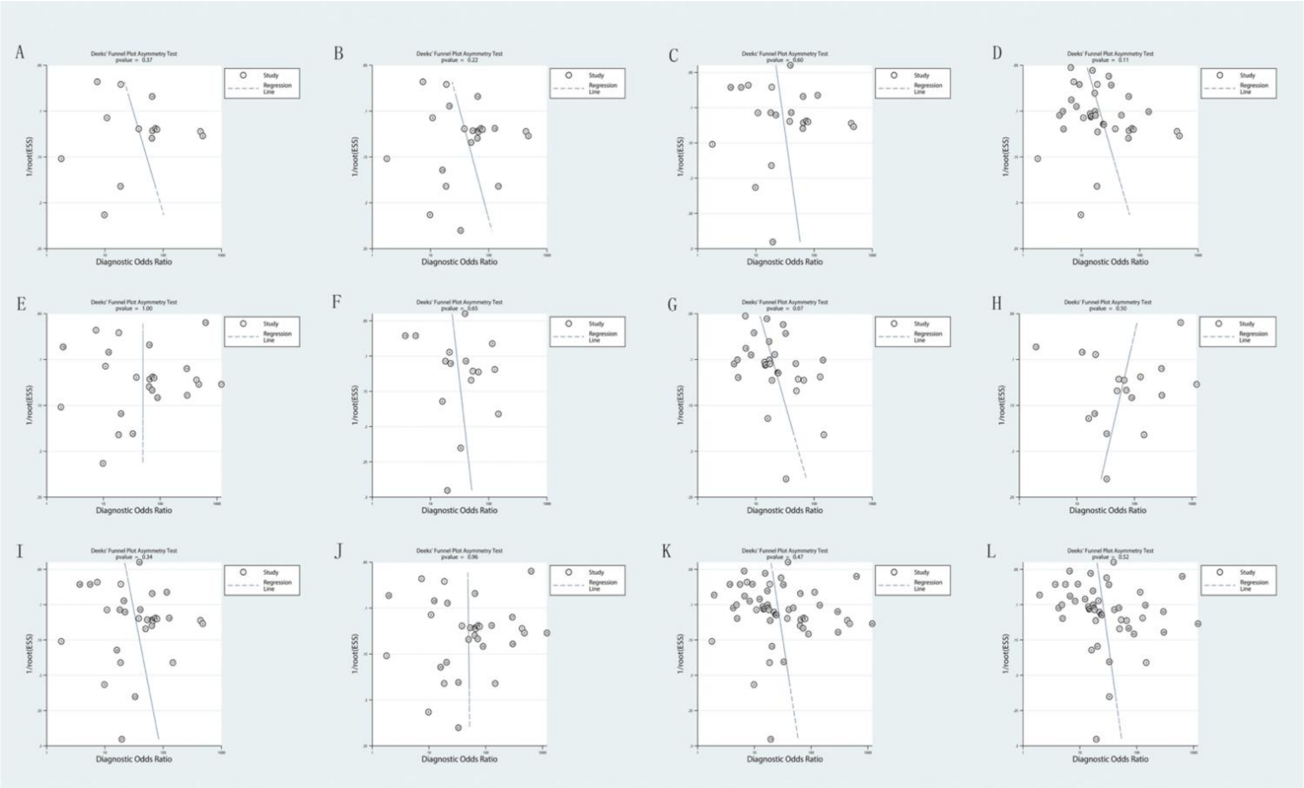

Supplementary Figure 3. Deek's funnel plot for the studies included in the meta-analysis.
